# Supplementary material for: Spin-torque skyrmion resonance in a frustrated magnet
Source: Nat Commun. 2025 May 18;16:4616. doi: 10.1038/s41467-025-59899-5 (PMC12086206; doi:10.1038/s41467-025-59899-5)
Supplement: Supplementary file 1 — Supplementary Information [file 41467_2025_59899_MOESM1_ESM.pdf]

Supplementary Information

Spin-torque skyrmion resonance in a frustrated magnet

Nirel Bernstein[1], Hang Li[2,3], Benjamin Assouline[1], Yong-Chang Lau[3,4], Igor Rozhansky[1,5], Wenhong Wang[2], Amir Capua[1]*

[1] Institute of Applied Physics, The Hebrew University of Jerusalem, Jerusalem 9190401, Israel
[2] School of Electronics and Information Engineering, Tiangong University, Tianjin 300387, China
[3] Beijing National Laboratory for Condensed Matter Physics, Institute of Physics, Chinese Academy of Sciences, Beijing 100190, China
[4] University of Chinese Academy of Sciences, Beijing 100049, China
[5] National Graphene Institute, University of Manchester, Manchester M13 9PL, United Kingdom

*e-mail: amir.capua@mail.huji.ac.il

Contents

Supplementary Note 1: Crystal structure characterization 3
Supplementary Note 2: Calculation of current density distribution 4
Supplementary Note 3: Calculation of the topological charge 5
Supplementary Note 4: Calculated texture in the presence of a DC STT 6
Supplementary Note 5: Magnetic textures as a function of H 7
Supplementary Note 6: Validation of the saturation conditions 8

Supplementary Note 1: Crystal structure characterization

X-ray diffraction measurements were carried out at room-temperature using a Bruker D2 machine. This data is presented in Fig. S1. A hexagonal surface is seen in the (001) plane.

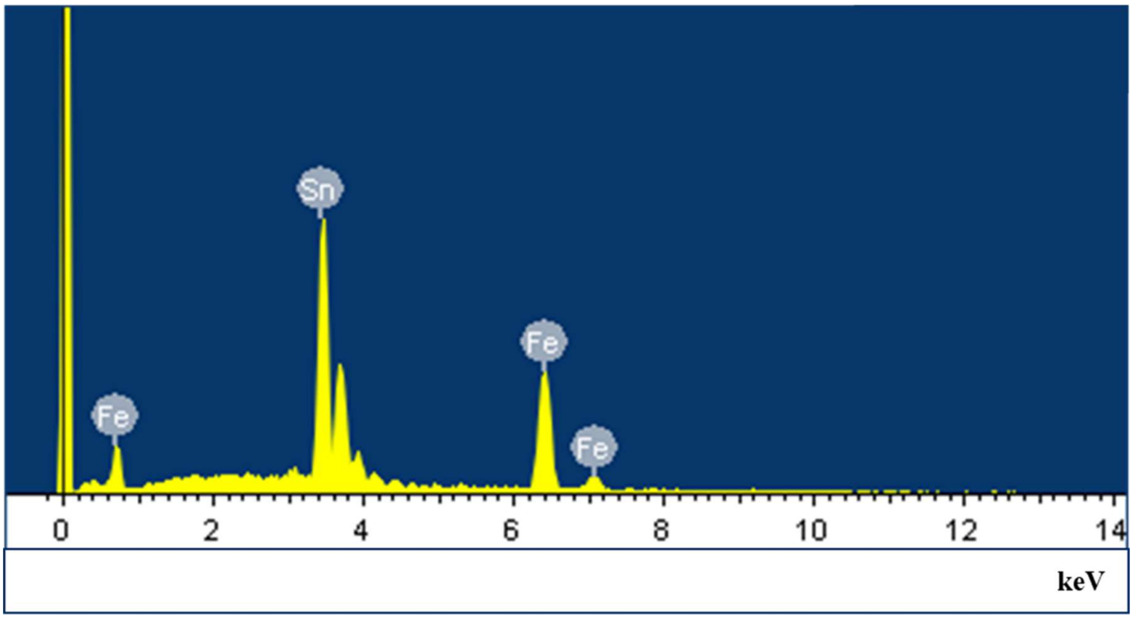

Figure S1. X-ray diffraction showing the formation of the Fe_3Sn_2.

Supplementary Note 2: Calculation of current density distribution

In the OSTFMR setup a current I_c was injected into the crystal. To convert between I_c to the current density, J_c, at the optically probed region, the Poisson equation was solved. To this end, the crystal dimensions and position of the electrical contacts were taken into account in a 3D model of the crystal as illustrated in Fig. S2. The location of the laser spot on the crystal was determined from a microscope image that was integrated into the OSTFMR setup. The Neumann boundary conditions were applied at the position of the electrical contacts. J_c was found to be homogeneous within several tens of μm away from laser spot both in the horizontal (depth) and lateral dimensions. Therefore, in the probed region, J_c and I_c were related by $J_c = A_0 \cdot I_c$. From the calculations, $A_0 = 3 \times 10^{11}\ m^{-2}$ was extracted.

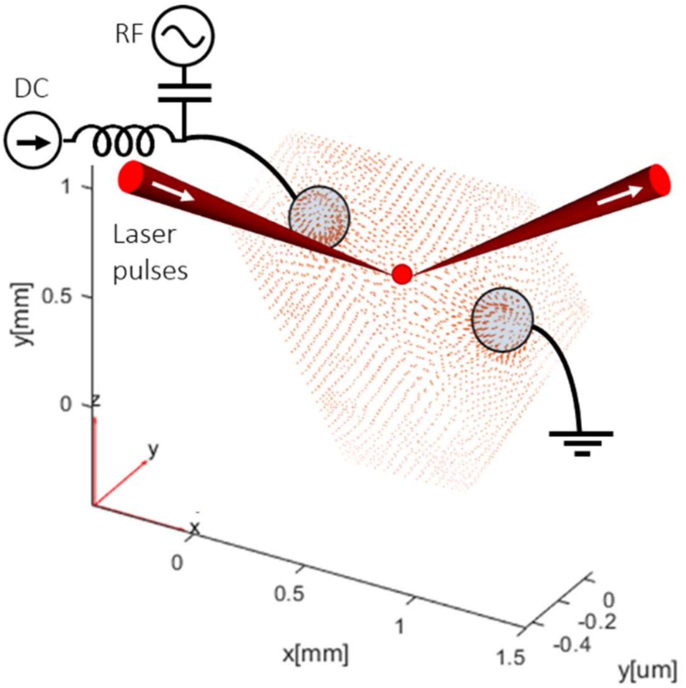

Figure S2. Current density calculations. The current density was calculated by solving numerically the Poisson equation in three dimensions. Black circles indicate position of contacts in the experiment and red vector lines represent the direction and magnitude of the current density. Red spot indicates position of the probing laser beam.

Supplementary Note 3: Calculation of the topological charge

The topological charge Q was calculated using the Berg-Luscher method [1]. The magnetic texture for the planar $\mu_0 H_0 = 250\,mT$ case is presented in Fig. S3(a). Figure S3(b) presents the topological charge density from which a trivial magnetic vortex was determined. In the OOP $\mu_0 H_0 = 250\,mT$ case, the calculation reveals that the topological texture is non-trivial. The texture is presented in Fig. S3(c). $Q = -1$ was determined from the topological charge density presented in Fig. S3(d). This behavior was also found experimentally [2].

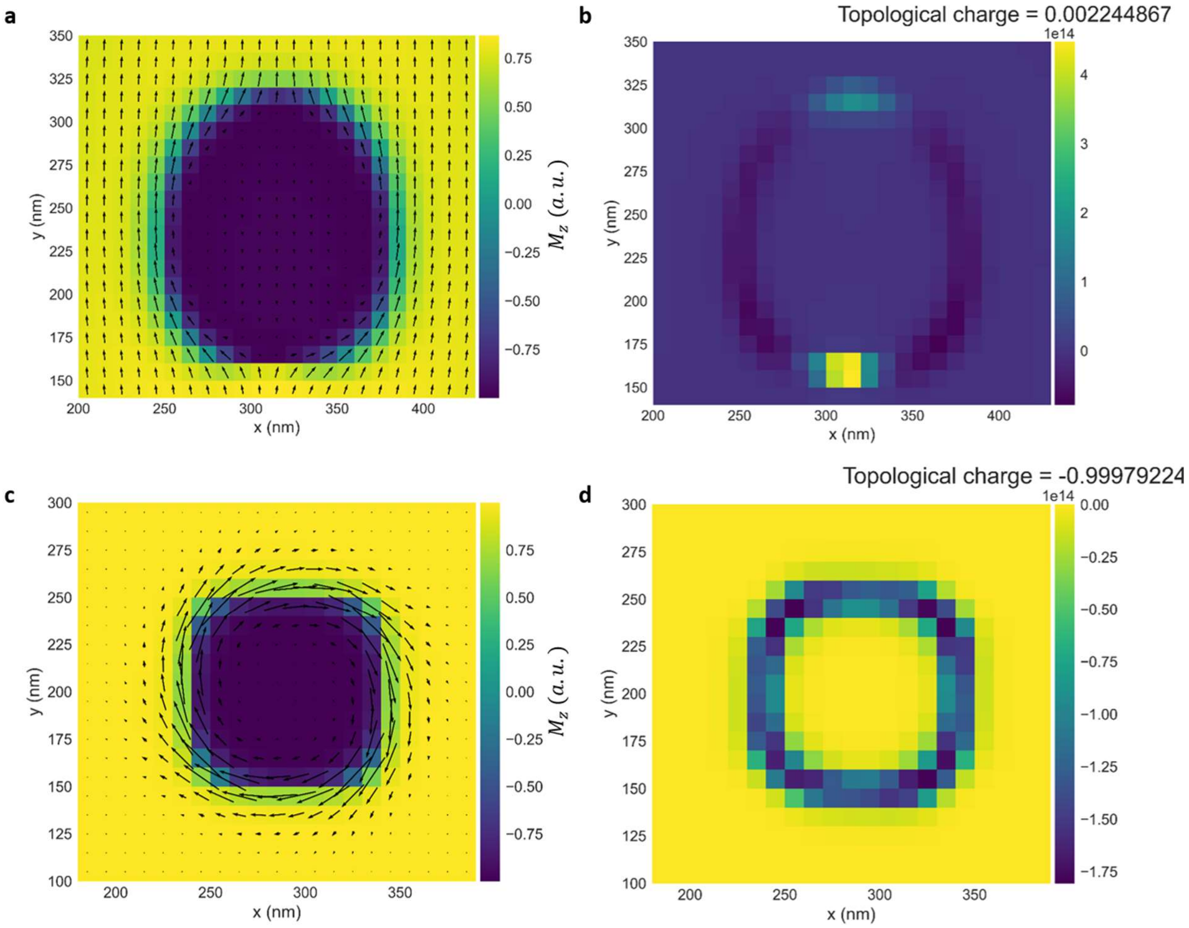

Figure S3. Comparison between IP and OOP textures at $\mu_0 H_0 = 250\,mT$. (a) Magnetic vortex calculated for IP H_0 at $\theta_H = 30°$. (b) Calculated topological charge density of the vortex presented in (a). A $Q = 0$ was evaluated illustrating that the texture is topologically trivial. (c) Magnetic skyrmion calculated for an OOP H_0. (d) Calculated topological charge density of the vortex presented in (c) resulting in $Q = -1$.

Supplementary Note 4: Calculated texture in the presence of a DC STT

The magnetic textures were calculated in the presence of a DC STT by incorporating the Zhang-Li term as described in the 'Methods' section. Figure S4 presents the calculated textures as a function of J_c for different IP magnetic field values, H_0. Except for the case of the disordered phase, it is seen that up to $J_c = 5 \cdot 10^{10}\ A\ m^{-2}$, which corresponds to the maximum value applied experimentally (highlighted by blue shading), the texture remains stable. In the case of the disordered phase having $H_0 = 0\ mT$, current induced texture switching takes place as indicated by the red frames. Overall, the critical current density required to observe STT-induced switching is an order of magnitude higher than the J_c applied experimentally.

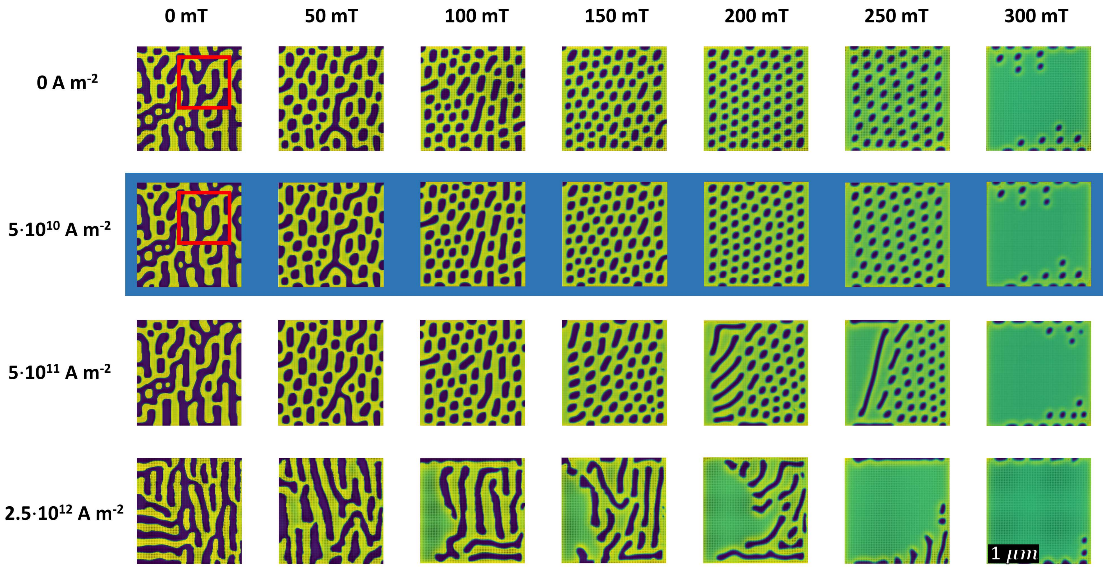

Figure S4. Magnetic textures in the presence of DC STT. Calculated textures as a function of J_c for different H_0 values. Blue highlighting indicates the maximum J_c applied experimentally. Red squares guide the eye to the regions where texture switching occurred.

Supplementary Note 5: Magnetic textures as a function of $\vec{H}$

The calculated textures as a function of IP $\vec{H}$ are presented in Fig. S5. The textures illustrate the crucial role of θ_H in the formation of the magnetic textures for the set of $\theta_H = 0°, 30°, 60°$, and $90°$ for which the PHE was measured.

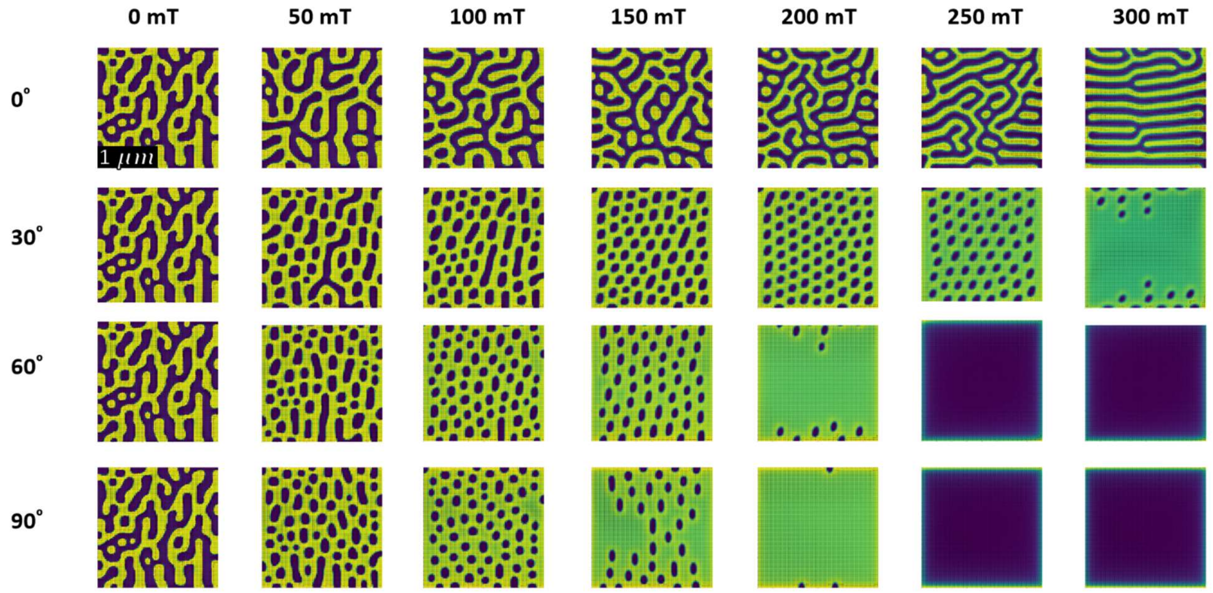

Figure S5. Magnetic textures as a function of IP $\vec{H}(H_0, \theta_H)$ for $\theta = 0°, 30°, 60°, 90°$.

Supplementary Note 6: Validation of the saturation conditions

Saturation conditions were verified by calculating M_z vs. H_0. M_z was determined from the mean $\hat{z}$ component of the magnetic textures as presented in Fig. S5. The non-monotonic behavior observed in the PHE measurements at $\theta_H = 30°, 60°$ and $90°$ is seen in Fig. S6(a), suggesting that $\rho_{xy}^{\parallel}$ stems mainly from the M_z-dependent k-space contribution. Furthermore, it is seen that M_z saturates to a different limit depending on θ_H. This feature stems from the tilted anisotropy axis. It is seen that the two cases of $\theta_H = 0°$ and $2°$ differ significantly. A peak appears already at $\theta_H = 2°$ showing that the peak observed in the measurement for $\theta_H = 0°$ stems from a slight misalignment. Figure S6(b) presents VSM measurement of the magnetization as a function of IP $\vec{H}$ from which a saturation field of $\sim 280\ mT$ was extracted.

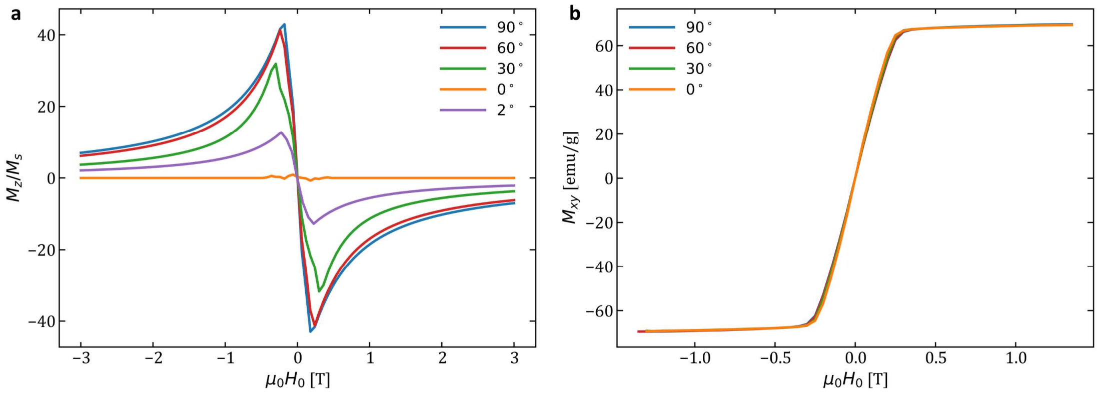

Figure S6. Calculated and measured M_z vs $\vec{H}$ as a function of θ_H. (a) Calculated M_z normalized to M_s as a function of IP H_0 revealing a non-monotonic behavior indicating the transition between magnetic phases. (b) VSM measurement of an IP magnetic field. Saturation occurs at $\mu_0 H_0 = 280\ mT$.

References

[1] B. Berg and M. Lüscher, ***"Definition and statistical distributions of a topological number in the lattice O(3) σ-model"***, Nuclear Physics B **190**, 412 (1981).
[2] Z. Hou, W. Ren, B. Ding, G. Xu, Y. Wang, B. Yang, Q. Zhang, Y. Zhang, E. Liu, F. Xu, W. Wang, G. Wu, X. Zhang, B. Shen, and Z. Zhang, ***"Observation of Various and Spontaneous Magnetic Skyrmionic Bubbles at Room Temperature in a Frustrated Kagome Magnet with Uniaxial Magnetic Anisotropy"***, Advanced Materials **29**, 1701144 (2017).
[3] S. Zhang and Z. Li, ***"Roles of nonequilibrium conduction electrons on the magnetization dynamics of ferromagnets"***, Physical Review Letters **93**, 127204 (2004).
